# The malarial blood transcriptome: translational applications

**DOI:** 10.1042/BST20230497

**Published:** 2024-02-29

**Authors:** Claire Dunican, Clare Andradi-Brown, Stefan Ebmeier, Athina Georgiadou, Aubrey J. Cunnington

**Affiliations:** 1 Section of Paediatric Infectious Disease, Department of Infectious Disease, Imperial College London, London, U.K.; 2 Centre for Paediatrics and Child Health, Imperial College London, London, U.K.

**Keywords:** diagnostics, malaria, prognosis, therapeutics, transcriptomics

## Abstract

The blood transcriptome of malaria patients has been used extensively to elucidate the pathophysiological mechanisms and host immune responses to disease, identify candidate diagnostic and prognostic biomarkers, and reveal new therapeutic targets for drug discovery. This review gives a high-level overview of the three main translational applications of these studies (diagnostics, prognostics, and therapeutics) by summarising recent literature and outlining the main limitations and future directions of each application. It highlights the need for consistent and accurate definitions of disease states and subject groups and discusses how prognostic studies must distinguish clearly between analyses that attempt to predict future disease states and those which attempt to discriminate between current disease states (classification). Lastly it examines how many promising therapeutics fail due to the choice of imperfect animal models for pre-clinical testing and lack of appropriate validation studies in humans, and how future transcriptional studies may be utilised to overcome some of these limitations.

## Introduction

Malaria is a mosquito borne febrile illness caused by apicomplexan parasites of the genus *Plasmodium*. Of the six species that most commonly cause human malaria, *Plasmodium falciparum* accounts for the great majority of clinical cases and deaths and is the focus of the current review. Infected individuals vary considerably in their disease trajectories and outcomes. At one end of the spectrum are those with completely asymptomatic infection, who are not classified as having malarial illness, but who represent the main reservoir of transmissible parasites in endemic regions [1]. Towards the other end of the spectrum are those who develop malaria symptoms, who start with a pre-symptomatic phase and then progress to symptomatic illness manifesting as uncomplicated malaria (UM) or severe malaria (SM). SM (defined by the presence of one or more clinical and laboratory features associated with increased risk of death) is usually described by a set of non-mutually exclusive clinical syndromes including cerebral malaria (CM, characterised by coma), severe malarial anaemia (SMA), and acidosis/hyperlactataemia [2].

Transcriptomics is the study of transcriptomes — the RNA in a biological system, such as a cell, tissue, or organism. Transcriptomic analyses may examine the expression of specific genes (targeted) or all the genes in a system (untargeted) in cross-sectional (single time-point) or longitudinal (multiple time points per subject) studies.

Transcriptomic studies have been used extensively in malaria to examine host response to infection, parasite pathogenic mechanisms, and the complex interactions between the two [3–5]. These studies have focused on single cells [6] and bulk tissues during natural infections [7] and also in controlled human malaria infection studies, where healthy volunteers are intentionally infected with malaria parasites and monitored under careful clinical supervision [8].

Blood is often the tissue of interest for malaria transcriptomic studies. This is because much host-pathogen interaction occurs within the blood, it is easily sampled, and transcriptomic changes within the blood may provide insights into the perturbed state of the rest of the body [9]. Often, whole blood (including leukocytes and infected and uninfected erythrocytes) is the sample of choice. It is debatable whether such studies should adjust for differences in the proportion of immune cell types between samples, which can influence the overall patterns of gene expression, because changes in immune cell populations are a fundamental part of the response to infection [10].

**Figure 1.**
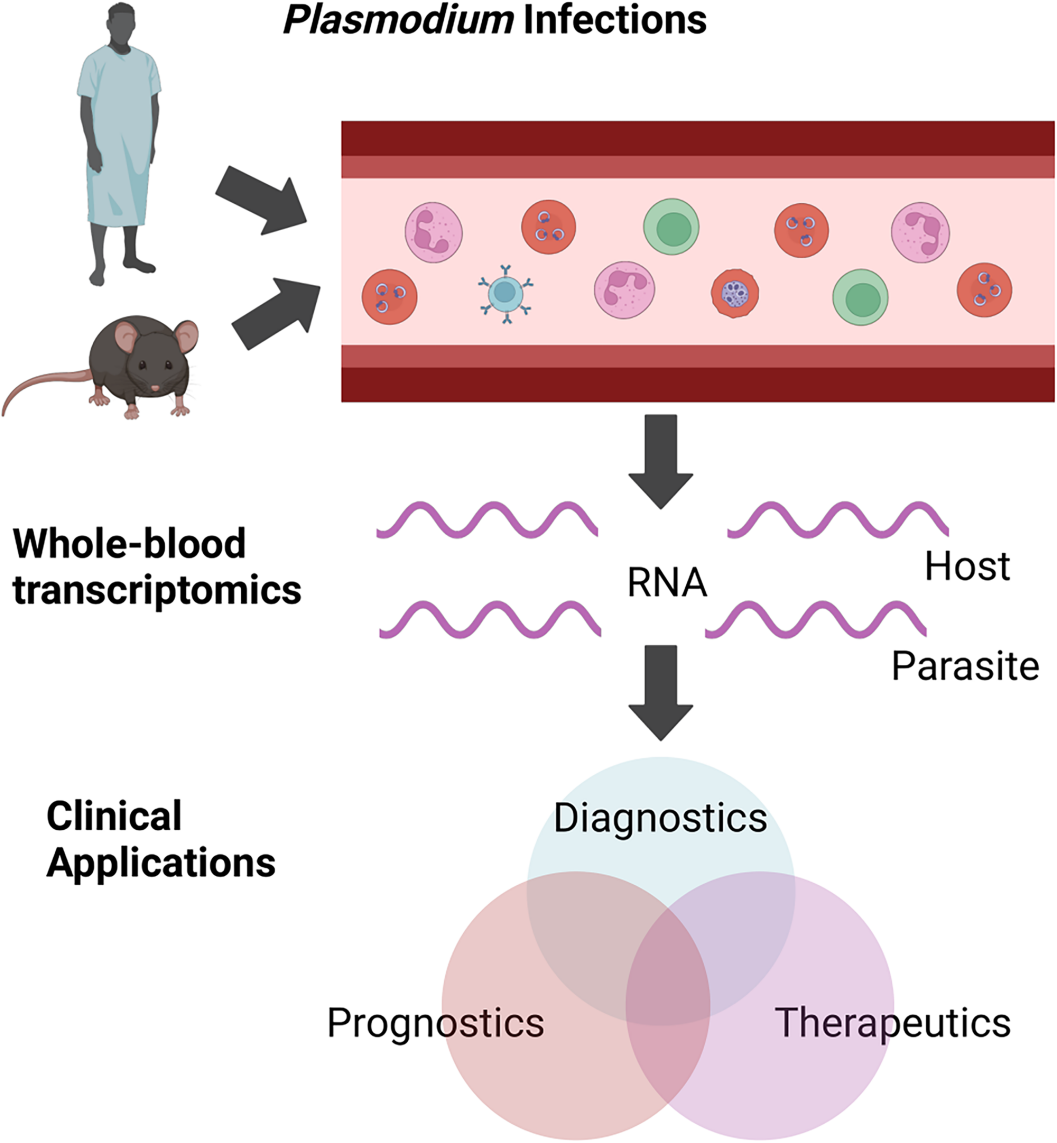
The translational applications of whole-blood transcriptomic studies of malaria. Whole blood (containing leukocytes, uninfected and infected erythrocytes) is collected from malaria parasite-infected subjects. RNA is extracted and quantified using RNA-sequencing or microarray and then bioinformatic analyses identify genes and biological pathways that could be used for improved diagnostics, prognostics, and therapeutics. Improvements in each of these three applications has potential to benefit development of improvements in the others. This figure was created with BioRender.com.

This review will give a high-level summary of the current and potential future translational applications of whole-blood transcriptomic studies in *P. falciparum* malaria, focusing on diagnostics, prognostics, and therapeutics (Figure 1). Potential translational applications include identifying host and pathogen biomarkers for diagnostic and prognostic tests as well as identifying new treatment targets for the various SM manifestations.

Though beyond the scope of the current review, blood transcriptomics may also have useful translational applications for non-falciparum malarias, for example identifying host signatures to distinguish between infection with different *Plasmodium* species and prediction of relapse in *Plasmodium vivax* and *Plasmodium ovale* infection.

## Diagnostics

Current diagnostic tools for malaria include the gold standard blood film microscopy, point-of-care antigen testing, and nucleic acid amplification methods including polymerase chain reaction (PCR) [11]. These tests rely on the direct detection of the parasite, its proteins, and its genetic material, respectively.

Microscopy is sensitive and specific for parasitaemia but requires basic laboratory facilities and a competent microscopist, limiting its use in many malaria-endemic settings. Point-of-care antigen testing is quicker and simpler to perform than microscopy, and is more widely available, but is vulnerable to false positives (recent but not current parasitaemia [12]) and false negatives (from parasites with *P. falciparum* histidine rich protein 2/3 — PfHRP2/3 deletions [12,13]). PCR testing, though more sensitive than microscopy, requires laboratory equipment and expertise not widely available in malaria endemic regions [14].

Even when the malaria parasite is detected, diagnosis is complicated by the phenomenon of host tolerance [11]. Individuals living in high transmission regions, who have had repeated exposure to malaria, often tolerate parasites in the blood without any symptoms (asymptomatic infection). Therefore, it may be challenging to determine whether a patient's febrile illness is due to the malaria parasites present in their blood or due to some other cause, such as bacteraemia, with incidental parasitaemia.

Overdiagnosis of malaria may be a considerable problem. For example, Watson et al. [15] used Bayesian latent class modelling of the relationship between platelet counts, PfHRP2, and the likelihood of severe disease, to estimate that up to one third of febrile African children diagnosed with SM may have another cause for their illness.

Rapid diagnostics capable of distinguishing between malaria mono-infection and bacterial coinfection are currently lacking. Such diagnostics may allow early identification of patients with bacterial coinfection for prompt administration of lifesaving antibiotics, which may be scarcer in resource-limited settings [16].

Therefore, although we already have sensitive and specific tests for detecting malaria parasites, the most accurate tests are often unavailable in malaria-endemic regions, and none help to make the important distinction between symptomatic vs incidental parasitaemia. Whole-blood transcriptome analysis may allow development of new diagnostic tools which overcome some of these limitations [17–19].

### Gene expression as the basis for diagnostic tests

Several different infectious and inflammatory diseases, which may be clinically indistinguishable at initial medical assessment, have demonstrated unique patterns of host gene expression. Comparison of the whole-blood transcriptome between groups of patients with different diseases has led to identification of gene expression signatures that could form the basis of future diagnostic tests [17–20].

Many whole-blood transcriptomic malaria studies have also identified differences in gene expression between UM and the various SM syndromes [3,4], as well as between malaria and other acute febrile illnesses [20,21]. Such differences in gene expression may form the basis for future diagnostic tests that distinguish between UM and SM, and between malaria and other causes of febrile illness.

Gene expression tests may also allow discrimination between symptomatic vs incidental parasitaemia. For example, Prah et al. [7] compared whole-blood gene expression in Ghanaian children with symptomatic malaria, asymptomatic parasitaemia, and healthy uninfected controls. Comparison with uninfected controls found many hundreds of differentially expressed genes in those with symptomatic malaria but no differentially expressed genes between the uninfected and those with asymptomatic parasitaemia.

In a longitudinal study of Malian children, Tran et al. [22] compared the blood transcriptome of asymptomatic infection with the same child's uninfected baseline and found some limited evidence of monocyte/activated dendritic cell induction in these children. Interestingly, when they performed the equivalent analysis on pre-symptomatic children, children who were asymptomatic at the time of sampling but subsequently developed symptoms, they detected no difference between the baseline and post-infection samples. This paired analysis of asymptomatic infection with each child's uninfected baseline increased power by accounting for inter-individual variation, thus allowing detection of smaller differences in gene expression.

Taken together, these results suggest that unlike symptomatic malaria, which triggers a broad transcriptomic response, asymptomatic malaria instead triggers minimal, sometimes undetectable, responses. The detection of this response may be dependent on sample timing, relative to the time of infection, a confounding factor that future transcriptomic analyses of human challenge experiments could overcome.

By utilising genes that distinguish unwell individuals with asymptomatic parasite carriage from those with malarial disease, future host-gene expression tests may reduce overdiagnosis of malaria and ensure the most suitable treatments are administered as swiftly as possible.

Gene expression tests also have the potential to discriminate simultaneously between multiple diseases with one blood sample and identify co-infections. While ‘one vs one’ and ‘one vs all’ disease comparisons may lead to gene expression tests that discriminate between two disease categories (malaria vs ‘not malaria’), tests capable of distinguishing between multiple diseases would be far more desirable. In an important proof-of-principle study, Habgood-Coote et al. [20] identified a 161-transcript signature which served to distinguish between 6 different disease classes (including malaria) with a single blood test, and malaria was particularly well discriminated using this approach. Tests such as this may allow for quicker diagnosis in the future, potentially saving lives by reducing the time to starting appropriate treatment.

To be useful in malaria-endemic settings, new diagnostic tests should be affordable, accurate, user friendly, give a rapid result, and should not require laboratory facilities or highly trained staff [23]. To meet these requirements for gene expression-based diagnostics, new technological innovations will be needed.

Pennisi et al. [24], recently described the development of a portable ‘lab-on-chip’ platform capable of testing blood samples for a two-gene RNA signature to discriminate between bacterial vs viral infection [24,25]. Platforms such as this may allow creation of point-of-care gene expressions tests providing more detailed and useful information than current point-of-care malaria antigen tests [26].

Overall, there are several theoretical advantages of using gene expression as the basis for new malaria diagnostics. First, these tests may allow discrimination between symptomatic vs incidental parasitaemia, thus reducing malaria overdiagnosis. Second, such tests may be capable of simultaneous discrimination between several diseases with a single blood sample [20], thus helping healthcare workers to make quick and accurate diagnoses. Third, the technology may be amenable to creating point-of-care tests that would be suitable for widespread use with minimal training [26].

Correct and timely diagnosis is clearly important in the clinical setting because it allows the patient to start appropriate treatment promptly. Correct diagnosis is also important in research, such as in selecting patients who genuinely have SM for clinical trials of new adjunctive therapies, and gene expression tests may also have a role in this context.

## Prognostics

The blood transcriptome of individuals with malaria may also be a useful basis for the prediction of future disease outcomes (prognosis). These outcomes could include progression to more severe disease (UM to SM), survival vs death, and development of long-term morbidity (such as neurological sequelae in SM patients).

To be useful to the patient, a malaria prognostic test would need to be affordable, accurate, and give a rapid result early during the illness, to identify patients at high risk for deterioration and adverse outcomes. Identifying patients with a poor prognosis may prompt closer monitoring and a lower threshold for treatment escalation with the aim of improving clinical outcomes. Conversely, identifying patients with very low risk of adverse outcome may allow them to be discharged from hospital and resources reallocated more efficiently.

The genes identified in blood transcriptomic studies may be translated into prognostic tests by identifying transcripts which distinguish between known future disease states [27], then using feature selection methods to reduce the number of transcripts for training and cross-validation. It is only with cross-validation and testing on independent ‘unseen’ data that the selected features (candidate transcript ‘signatures’) may be truly validated as predictive [28]. Such signatures also require validation using alternative methods of measuring gene expression, such as reverse transcription-PCR and loop-mediated isothermal amplification, which are more suited to molecular point-of-care tests [24,29]. This would ensure that the signature can be translated onto devices that can be used in clinic to swiftly aid treatment and triage decisions.

There are currently no prognostic blood gene signatures in clinical use for malaria patients, although a variety of clinical scoring systems exist [29,30], such as the ‘Malaria Prognosis Score’ [31] and Lambaréné Organ Dysfunction Score [32].

### Transcriptomic prognostic studies

A few studies have examined the prognostic value of the blood transcriptome in malaria. For example, Tran et al. [22] used random forest models on uninfected baseline samples to predict febrile malaria at or after incident parasitaemia in Malian children. In a different longitudinal study from Mali, Mbambo et al. [33] identified genes differentially expressed at baseline between children who went on to develop symptomatic malaria during the malaria season and those who did not.

Many studies using transcriptomics to classify subjects based on current disease severity have claimed prognostic value and mention how genes are associated with disease ‘progression’ but have not formally evaluated prediction of future outcomes [3,34,35]. This has led to some confusion in the literature about the prognostic potential of transcriptomics, although classifying subjects as severe or non-severe does have intrinsic prognostic value because SM, by definition, carries an increased risk of death or disability [34,36].

Prognostic transcriptomic signatures have been developed for other diseases. Liu et al. [37] used differential expression analysis, random forest, and paired gene profile analysis to identify three 10-gene signatures to predict death vs survival in Ebola virus disease patients. Lei [38] identified the prognostic potential of a single transcript measured at the time of hospitalisation to predict future severity of Covid-19. However, to date most of the true ‘prognostic’ biomarker studies in malaria have focussed on soluble (plasma or serum) host or parasite proteins, rather than transcriptomic signatures [30].

### Genes and pathways associated with severity of illness

Previous transcriptomic studies have identified many hundreds of human and parasite genes differentially expressed between UM and SM [5,39–47]. Common findings include up-regulation of neutrophil and granulopoiesis related genes, interferon-gamma related genes, and type-1 interferon signalling genes in SM [5,21,44]. However, Karikari et al. [44] found that neutrophil activation was unique to CM compared with other non-cerebral SM phenotypes. Lee et al. [5] found that up to 99% of human expression differences between different clinical manifestations of malaria were driven by variation in parasite load. Conversely, parasite gene expression did not show the same dependence.

The polymorphic *var* gene family (which codes for *P. falciparum* Erythrocyte Membrane Protein 1 — PfEMP1) is arguably the most well-known virulence-associated multi gene family of *P. falciparum*. Despite the high level of polymorphism, expression of specific subsets of the *var* gene family consistently associate with specific disease manifestations, with a more conserved *var* transcriptome thought to be associated with severe disease [34,42,45]. Furthermore, the levels of *var* transcripts with specific PfEMP1 predicted binding phenotypes have displayed consistent associations with both UM, SM, and subtypes of SM [48,49]. For example, Shabani et al. [49] observed increasing severity of malaria was associated with increasing levels of EPCR-binding PfEMP1 transcript levels, with the highest levels observed in children with CM and SMA. This implies *var* gene expression may predict both severity and SM syndrome. There is also intriguing evidence to suggest that reduced expression of *var* genes may play a role in long lasting asymptomatic chronic infections [50].

Previous studies have been restricted by their *var* gene characterisation approach [51,52]. The extreme polymorphism observed in these genes essentially means each parasite strain has a unique repertoire of *var* genes, making them very complex to accurately characterise. Recent approaches for characterising *var* genes from RNA-sequencing open the way for their prognostic potential to be better quantified [45,53].

### Challenges for prognostic studies

Due to the relatively high cost of generating transcriptomic data, most studies to date have only modest sample sizes. This is problematic because the relative rarity of death or disability mean large sample sizes would be required for enough of these events to be observed in a prospective cohort.

In addition, malaria blood transcriptomic studies to date have had insufficient samples sizes (usually far fewer than 100 subjects per class) for performing adequate training and cross-validation followed by testing on an independent ‘unseen’ data set (the latter is needed for prognostic inference). Even when cross-validation is used for the whole dataset, the risk of overfitting and instability is high, making it important to, as a minimum, iterate the cross-validation over multiple runs to evaluate the stability of the predictive features. Additionally, models that require the optimisation of pre-defined hyperparameters should consider the use of nested cross-validation for model training to improve the generalisation of results. Therefore, there is a general need for malaria transcriptomic datasets to be larger, and perhaps more standardised to allow for comparison between different studies.

Furthermore, it is necessary to validate prognostic models on independent datasets to understand their generalisability. Many prognostic models and biomarkers also lack this appropriate validation in different disease settings and populations, which would be required for a prognostic test to enter widespread clinical use. Many of these studies use samples from one geographical region, i.e. sub-Saharan Africa [42,46,54], but it is often unknown whether their results can be extrapolated to other areas due to differences in the transmission intensity and disease classification. This is important because SM manifestations vary with the epidemiology of malaria transmission [55].

More widespread adoption of the ‘Transparent Reporting of a multivariable prediction model for Individual Prognosis or Diagnosis’ guidelines, specifically the inclusion of a clear statement of which features are being predicted, should improve the transparency of reporting prediction models and reduce the aforementioned diagnostic-prognostic ambiguity in the literature [56].

Prognostic studies may also be confounded by the medical treatment given to a patient after sampling, with some subjects surviving when they would otherwise have died, and possibly some dying when they would otherwise have survived (as observed in the ‘Fluid Expansion as Supportive Therapy’ trial) [57–61]. The effect of treatment would not be reflected in the pre-treatment blood gene expression and may make any prognostic signatures appear to perform poorly. Following this, because patients often present to hospital only when they are already severely ill, the early course of their infection is not observed so identifying subjects for transcriptomic studies that examine progression from mild disease is challenging.

To increase their robustness and statistical power, prognostic studies need to start with well-defined patient groups, where the true cause of illness is known [62]. Therefore, prognostic studies in malaria could be substantially improved by new diagnostic methods to distinguish unwell patients whose illness is due to malaria alone from those with incidental parasitaemia and another cause of severe illness. Thus, transcriptomic approaches could improve both the design of prognostic studies and provide new methods for predicting adverse outcomes.

## Therapeutics

Therapeutics are substances administered to treat or prevent disease. There are many different types of anti-malarial therapeutic, including artemisinin-based combination therapies, often used to treat uncomplicated *P. falciparum* malaria, and intravenous artesunate and quinine, often used to treat SM [63–65]. Unfortunately, the increasing incidence of anti-malarial drug resistance [66,67] means that new malaria therapeutics may be needed in the near future.

Adjunctive therapies are treatments administered in addition to those directly targeting the pathogen, with the aim of improving disease outcomes — they may also be described as host-directed therapies and may assist pathogen elimination, reduce tissue damage, or promote recovery. Most candidate adjunctive therapies for SM have shown no benefit or were even found to be harmful [68], although regular paracetamol administration has recently shown some promise in reducing the risk of acute kidney injury in SM [69].

The motivation of many whole-blood transcriptomic studies is to identify new (host and parasite) therapeutic targets (proteins or pathways) that are associated with pathophysiological mechanisms of severe illness. Once targets of interest are identified in human studies, transcriptomics can be used to select the most appropriate animal models in which to test them, select subjects for inclusion into clinical trials as well as improve our understanding of the biological mechanisms of pre-existing therapeutics and determine why individuals respond differently to treatment.

### Animal models

New therapeutics are often first tested in animal models. However, these models must accurately recapitulate pathogenesis of human malaria phenotype(s). Selection of inappropriate animal models is one possible cause of promising drugs failing at human trial stage [70]. Recently, comparative transcriptomic approaches have been used to identify translationally relevant mouse models for different human malarial phenotypes including CM, SMA, and hyperlactatemia [71], reasoning that the transcriptome may reflect many of the biological processes associated with disease. This approach could be used to identify animal models in which there is concordance of biological processes relevant to the specific therapeutic target and treatment between the mouse model and human disease, maximising the chance that results from the mouse model will be translatable to humans.

### Trial participant selection

The challenge of distinguishing incidental parasitaemia from ‘true’ malaria in sick patients has important implications for clinical trials of adjunctive treatments. The power to detect beneficial effects of treatment may be greatly diminished if a substantial proportion of study participants have another cause for their severe disease. The recent evidence that up to one third of subjects in some large studies of SM may be misclassified [15] could be another reason why many adjunctive treatment trials did not show benefit. Improved diagnostics based on gene expression signatures may refine the identification of participants with true malarial disease for future clinical trials, improving their ability to accurately assess both future and currently available therapeutics.

### Biological mechanisms of established therapeutics

Transcriptomic studies have also helped elucidate the biological mechanisms of existing drugs. For example, parasite transcriptomic studies have contributed to our understanding of drug-resistance and how this may be combatted with future therapeutics [72,73]. Mok et al. [73] investigated the molecular mechanisms of artemisinin resistance *in vivo* in 1042 *P. falciparum* parasites from patients with acute infections and identified the unfolded protein response as associated with protection against artemisinin. Antony et al. [72] compared the whole-blood transcriptomic profiles of chloroquine-resistant and chloroquine-sensitive *P. falciparum* strains and identified many potential therapeutic targets associated with immune-evasion and pathogenesis including cell surface-antigens.

### Individual treatment response variation

Individual patients often have different responses to malaria treatment despite similar status at the start of treatment. This may be due to a combination of host [74,75] and parasite genetic factors (drug-resistance genotype), and previous disease exposure. Future studies could stratify patients based on their response to malaria treatments, use transcriptomics to understand the underlying biology and identify those most likely to respond well to new treatments, opening the possibility of personalised medicine for malaria. Developing new treatments for malaria is costly and time-consuming, and new treatments often fail at the animal or clinical trial stages. This failure rate could be reduced by using transcriptomic studies to improve diagnostics, inform animal model selection, and instruct personalised treatment decisions. There should also be greater efforts to increase the comparability between studies by improving consensus around study design.

## Conclusion

Blood transcriptomics is a promising basis for diagnostic and prognostic signatures to improve management for patients with malaria. The whole-blood malaria transcriptome also has the potential to identify novel therapeutic targets, and to guide improved approaches to testing of new therapeutics. However, there are many limitations of current studies which need to be overcome to realise the potential of transcriptomics for translational applications. Amongst these, ensuring adequate sample sizes, well-defined disease groups, and appropriate study design for prognostic studies, will be particularly important to enable us to fully harness the power of the blood transcriptome in malaria.

## Perspectives

- Malaria is one of the most important infectious diseases affecting humanity today, estimated to cause more than 200 million cases and more than 600 000 deaths annually. To improve patient care and reduce the global burden of malaria, new diagnostic, prognostic, and therapeutic tools are needed.
- Analysis of the malaria blood transcriptome may identify gene expression signatures that could form the basis of new diagnostic and prognostic tests, reveal novel targets for new adjunctive (host-targeted) therapies, and help select the most appropriate animal models for testing new treatments.
- New diagnostics based on host gene expression may distinguish malaria from other diseases accompanied by incidental parasitaemia, improving the specificity of the definition of malaria. This would also underpin advances in prognostics and therapeutics, which are dependent on the correct subjects being studied.

## Open Access

Open access for this article was enabled by the participation of Imperial College London in an all-inclusive *Read & Publish* agreement with Portland Press and the Biochemical Society under a transformative agreement with JISC.

## Abbreviations

CM: cerebral malaria
PCR: polymerase chain reaction
SM: severe malaria
SMA: severe malarial anaemia
UM: uncomplicated malaria
